# Isolation, Enrichment and Analysis of Aerobic, Anaerobic, Pathogen-Free and Non-Resistant Cellulose-Degrading Microbial Populations from Methanogenic Bioreactor

**DOI:** 10.3390/genes16050551

**Published:** 2025-04-30

**Authors:** Lyudmila Dimitrova, Yana Ilieva, Dilnora Gouliamova, Vesselin Kussovski, Venelin Hubenov, Yordan Georgiev, Tsveta Bratanova, Mila Kaleva, Maya M. Zaharieva, Hristo Najdenski

**Affiliations:** 1 Department of Infectious Microbiology, The Stephan Angeloff Institute of Microbiology, Bulgarian Academy of Sciences, 1113 Sofia, Bulgaria; illievayana@gmail.com (Y.I.); vkussovski@gmail.com (V.K.); y.georgiev001@gmail.com (Y.G.); tsveta_bratanova@abv.bg (T.B.); milakalevavet@abv.bg (M.K.); zaharieva26@yahoo.com (M.M.Z.); hnajdenski@abv.bg (H.N.); 2 Department of General Microbiology, The Stephan Angeloff Institute of Microbiology, Bulgarian Academy of Sciences, 1113 Sofia, Bulgaria; dilnorag@gmail.com; 3 Department of Biotechnology, The Stephan Angeloff Institute of Microbiology, Bulgarian Academy of Sciences, 1113 Sofia, Bulgaria; vhubenov@microbio.bas.bg

**Keywords:** microbial consortia, biodegradation of cellulose, antimicrobial resistance, long-term storage, PCR, metagenomic analysis

## Abstract

**Background:** Nowadays, the microbial degradation of cellulose represents a new perspective for reducing cellulose waste from industry and households and at the same time obtaining energy sources. **Methods:** We isolated and enriched two aerobic (at 37 °C and 50 °C) and one anaerobic microbial consortium from an anaerobic bioreactor for biogas production by continuous subculturing on peptone cellulose solution (PCS) medium supplemented with 0.3% treated or untreated Whatman filter paper under static conditions. Samples were taken every 7 days until day 21 to determine the percentage of cellulose biodegradation. We determined the antimicrobial resistance of aerobic and anaerobic consortia and some single colonies by disc diffusion method, against 42 clinically applied antibiotics. PCR analyses were performed to search for the presence of eight genes for cellulolytic activity and nine genes for antibiotic resistance. By metagenomics analysis, the bacterial and fungal genus distributions in the studied populations were determined. **Results:** Aerobes cultured at 50 °C degraded cellulose to the greatest extent (47%), followed by anaerobes (24–38%) and aerobes (8%) cultured at 37 °C. The bacterial sequence analysis showed that the dominant phyla are *Bacillota* and *Bacteroidetes* and genera—*Paraclostridium*, *Defluvitalea*, *Anaerobacillus*, *Acetivibrio*, *Lysinibacillus*, *Paenibacillus*, *Romboutsia*, *Terrisporobacter*, *Clostridium*, *Sporanaerobacter, Lentimicrobium,* etc. in a different ratio depending on the cultivation conditions and the stage of the process. Some of these representatives are cellulolytic and hemicellulolytic microorganisms. We performed lyophilization and proved that it is suitable for long-term storage of the most active consortium, which degrades even after the 10th re-inoculation for a period of one year. We proved the presence of *ssr*A, *ssr*A BS and *bla*TEM genes. **Conclusions:** Our findings demonstrated the potential utility of the microbial consortium of anaerobes in the degradation of waste lignocellulose biomass.

## 1. Introduction

Nowadays, waste lignocellulosic biomass is one of the biggest environmental problems because it takes up valuable space in landfills. However, it can also serve as a renewable carbon resource for the production of chemicals like carboxylic acids (formic, acetic and succinic acids) and other value-added products like energy (biofuels) [1,2,3]. The textile industry uses approximately 8000 chemicals, including dyes, of which 30 cannot be eliminated by waste treatment and 72 are hazardous and contaminate surface and groundwater resources [4]. The Waste Framework Directive (2008/98/EC) introduces a new procedure for defining end-of-waste criteria [5] and in 2023 revision of the Directive focuses on textile waste [6]. As we all know, textile materials contain a high percentage of cotton, which is also involved in the production of textiles and medical products (gauze, bandages, filter paper, etc.) [7] and other cellulosic materials. Cotton fibers contain approximately 95% cellulose, whereas lignocellulosic biomass contains only 35–50% cellulose [8]. Solid organic cellulose particles from wastewater can be filtered and subjected to biological processing to produce biomethane, which can be used as a source of heat, steam and electricity and can be further processed into vehicle fuel or for the production of chemicals and/or proteins. It can also be used as a household fuel or in fuel cells [9,10,11]. Cellulose is a polysaccharide composed of repeating units of D-glucose linked by β-1,4-glycosidic bonds and it is found in all higher plants as a structural material in cell walls. It is a highly insoluble polymer [12]. Approximately 70% of organic waste containing cellulose is biodegradable. Biological methods for its degradation include microbial fermentation and enzymatic hydrolysis to depolymerize lignocellulosic polysaccharides [13,14]. In addition to cellulose, lignocellulosic materials are known to contain lignin and hemicellulose. Like cellulose, hemicellulose is a macromolecule made up of various sugars, whereas lignin is an aromatic polymer made from precursors of phenylpropanoid [15]. The process occurs either extracellularly or in association with the cell wall layer. There are two kinds of extracellular enzyme systems used by microorganisms. The first type of system is hydrolytic, which generates hydrolases to degrade cellulose and hemicellulose. The second is an oxidative and extracellular ligninolytic system that breaks down lignin. [15]. To function, these enzyme systems must be stable in the environment and resistant to the action of proteolytic agents [15,16].

Recently, many authors have isolated microbial communities that degrade cellulose from different habitats, such as water [17]; soil [17,18,19,20]; fertilizer, cow dung and waste effluent [19]; invertebrates (termite, snail, caterpillar and bookworm [21], hindgut of *Holotrichia parallela* larvae [22]); groundnut residues, rice straw and rotten wood [23]; decayed corn straw [20], etc. There are reports in the literature about bacterial species that can use cellulose as a carbon source, primarily by producing cellulase [19]. Such species belong to the genera *Corynebacterium*, *Clostridium (C. thermocellum*, *C. cellulolyticum*, *C. cellulovorans and C. josui)*, *Cellulomonas*, *Pseudomonas*, *Actinomyces*, *Ruminococcus (R. albus)*, *Fibrobacter (F. succinogenes),* as well as *Thermobifida fusca* [24,25]. The following fungal genera were studied for their cellulolytic enzymes and/or wood-degrading capability: *Bulgaria*, *Chaetomium and Helotium (Ascomycetes); Coriolus*, *Phanerochaete*, *Poria*, *Schizophyllum and Serpula (Basidiomycetes)*; *and Aspergillus*, *Cladosporium*, *Fusarium*, *Geotrichum*, *Myrothecium*, *Paecilomyces*, *Penicillium and Trichoderma (Deuteromycetes)* [25], incl. the species *Trichoderma viride* [26], *Trichoderma reesei* [26,27], *Trichoderma koningii* [28] and *Phanerochaete chrysosporium* [29], etc. The group of cellulase enzymes includes endoglucanase, exoglucanase and β-glucosidase, which act synergistically to completely degrade cellulose into monosaccharide units by breaking down β-1,4-glycosidic bonds [30]. Bacterial cellulases are characterized by high specific activity and stability over high temperature and pH, compared to fungal ones [31,32]. In addition, it is known that bacteria have a higher growth rate than fungi, is resistant to adverse environmental conditions, possess a high level of cellulase synergy, etc., which makes them preferred for various sectors in the industry, such as food, textile, paper, green chemistry, etc. [32,33,34,35]. Microbial consortia are preferable to single strains because microorganisms in a population act synergistically through their cellulases, have high metabolic diversity, and adapt more easily to environmental changes, leading to overall improved productivity. On the other hand, working with microbial consortia is significantly more difficult, as appropriate species must be selected, interspecific interactions must be coordinated, and community activity must be maintained over the long term [3,19,36].

It should be emphasized that, to date, no research team has attempted to achieve 100% biodegradation. This is one of the reasons why more and more researchers, including our team, are working in the area of microbial degradation of cellulose by developing specific approaches for a fast and efficient process. Research is focused on the optimization of key parameters for the growth of microorganisms and the optimization of culture conditions according to the substrates used [37]. In this study, we aimed to isolate and enrich cellulose-degrading microorganisms from a working methanogenic bioreactor under different culture conditions to achieve consortia with high cellulolytic activity and to assess their antibiotic resistance.

After searching, some of the genes related to antibiotic resistance and cellulose biodegradation were validated. Furthermore, genus diversity was estimated by metagenomics analysis.

## 2. Materials and Methods

### 2.1. Methanogenic Bioreactor

The source of microorganisms used in the present study was a digestate taken from an anaerobic laboratory-scale bioreactor for biogas production. The work volume of the bioreactor was 15 dm^3^ and it is operated in once-a-day feeding mode. The bioreactor was equipped with a DC motor for continuously stirring and automated temperature control using a temperature sensor (Pt 100), silicone rubber flexible heating pad and temperature controller to maintain the temperature in the bioreactor at 35 ± 0.5 °C. The process was realized using alkali pre-treated wheat straw as a raw material at OLR = 0.9 g VS/dm^3^/day.

### 2.2. Isolation and Enrichment of Microbial Consortia

Before each experiment, a fresh peptone cellulose solution (PCS) medium (0.5% peptone (Merck Milipore, 70169, Burlington, MA, USA), 0.1% yeast extract (Difco, 0127, Detroit, MI, USA), 0.15% CaCO_3_ (Merck Milipore, 102064, Burlington, MA, USA), 0.5% NaCl (Merck Sigma-Aldrich, S5886, Saint Louis, MI, USA) supplemented with 0.3% pretreated filter paper powder or untreated filter paper in pieces (1 × 1 cm) as an indicator of cellulase activity [22] were prepared.

In experiments with the disc-diffusion test, 1.5% of agar was added to the PCS medium. The probe from the methanogenic bioreactor was cultured in different conditions: aerobic at 37 °C, aerobic at 50 °C and anaerobic conditions in jars by gas-generating GasPak^TM^ EZ bags (Becton, Dickinson and Company, 260678, Sparks, MD, USA) at 37 °C. The anaerobic condition of the medium was validated using Dry Anaerobic Indicator Strips (Becton, Dickinson and Company, 271051, USA).

### 2.3. Determining the Degree of Filter Paper Degradation

All three enriched microbial consortia were used to determine their cellulolytic activity. Each probe (12 mL) was inoculated into a 50 mL PCS medium supplemented with 0.3% pretreated or untreated filter paper (visual control). Every 7 days for 28 days, 8 mL samples were taken from the flask with powdered filter paper to determine cellulose degradation according to the protocol of Updegraff (1969) [38]. The method is colorimetric (620 nm), simple, rapid and allows determination of cellulose biodegradation. The cellulose residues were extracted with acetic nitric reagent to remove lignin, hemicellulose and xylosans. A final solution of 67% H_2_SO_4_ was added to each sample. Cellulose was determined by the anthrone method.

When visible degradation of the filter paper pieces in the culture medium was observed, the consortia were then transferred to new flasks containing powdered filter paper. Samples were taken periodically (every 7 days for 28 days) to quantify the percentage of degraded cellulose.

### 2.4. Antimicrobial Test

Antimicrobial susceptibility testing was performed via the standard disk diffusion method (the Kirby–Bauer method) according to the protocols of the CLSI [39]. A broad panel of antibiotics were used, and most of the antibiotic disks had quantity of antibiotic according to EUCAST [40]. The antibiotics amoxicillin (25 µg, SD129-1CT), ampicillin (10 µg, SD002-1CT), carbenicillin (100 µg, SD004-1CT), oxacillin (5 µg, SD043-1CT), penicillin G (10 units, SD028-1CT), piperacillin (30 µg, SD066A-1CT), piperacillin/-tazobactam (30–6 µg, SD292E-1CT), ticarcillin (75 µg, SD074-1CT), ticarcillin/clavulanic acid (75/10, SD201-1CT), doxycycline HCl (30 µg, SD012-1CT), tetracycline (30 µg, SD037-1CT), cefamandole (30 µg, SD200-1CT), ceftazidime (10 µg, SD062A-1CT), cefepime (30 µg, SD219-1CT), aztreonam (30 µg, SD212-1CT), ciprofloxacin (5 µg, SD060-1CT), pefloxacin (5 µg, SD070-1CT), levofloxacin (5 µg, SD216-1CT), nalidixic acid (30 µg, SD021-1CT), gentamycin (10 µg, SD016-1CT), streptomycin (10 µg, SD031-1CT), tobramycin (10 µg, SD044-1CT), vancomycin (5 µg, SD155-1CT), erythromycin (15 µg, SD013-1CT), clarithromycin (15 µg, SD192-1CT), chloramphenicol (30 µg, SD006-1CT), colistin (methanesulfonate, 10 µg, SD009-1CT), novobiocin (5 µg, SD121-1CT), trimethoprim/sulfamethoxazole (co-trimoxazole, 1.25–23.75 µg, SD010-1CT) were provided from HiMedia (Mumbai, India). The antibiotics amoxicillin/clavulanic acid (20–10 µg, AUG30C), ceftazidime-avibactam (10–4 µg, CZA14C), ceftolozane/tazobactam (30–10 µg, C/T40), doripenem (10 µg, DOR10C), imipenem (10 µg, IMI10C), meropenem (10 µg, MEM10C), were from Mast Group Ltd. (Bootle, UK) The antibiotics doxycycline (30 µg), amikacin (30 µg), kanamycin (30 µg), rifampin (5 µg), bacitracin (0.07 E), lincomycin (15 µg), were from Bul Bio Ltd. (NCIPD EAD, Sofia, Bulgaria). The results were evaluated according to the cut-off breakpoint values of Manual of BBL Products and Laboratory Procedures [41] and the EUCAST [40], where applicable.

### 2.5. Long-Term Storage by Lyophilisation

We performed lyophilisation of 10 mL inoculum after centrifugation (5000 rpm for 5 min) in 250 µL 10% SM (HiMedia, GRM1254, Mumbai, India) in a vertical freeze dryer (BIOBASE Group, BKFD18P, Jinan, China) for long-term storage in serum vials of 10 mL volume (WHEATON, 6521251, Millville, NJ, USA) with 3-leg rubber stopper (WHEATON, 4663296, Millville, NJ, USA). The vials were covered with aluminium caps (WHEATON, 6252785, Millville, NJ, USA) by crimping (Fisherbrand™, 11550525, Schwerte, Germany).

### 2.6. Isolation of rRNA

Total rRNA from the aerobic, anaerobic and lyophilized anaerobic actively degrading cellulose culture was isolated by GeneMATRIX Universal RNA Purification Kit (EURx Ltd., E3598, Gdańsk, Poland). Concentrations of rRNA were measured on a Thermo Scientific NanoDrop Lite Spectrophotometer (Temecula, CA, USA) and standardized according to the manufacturer’s protocol. We performed reverse transcription with iScript™ Select cDNA Synthesis Kit (Bio-Rad Laboratories, Inc., 170-8896, Hercules, CA, USA).

### 2.7. Conventional PCR

For detection of cellulose-specific genes (*Cbp*A, *Eng*H, *Eng*M, *Eng*E, *Exg*S, *Ssr*A BS, *Ssr*A and *Xyn*A) and genes for antimicrobial resistance (ESBLs-TEM, *Qnr*A, *Qnr*B, *Qnr*B60, *Aac*(3)-IV, *Bla*SHV, *Bla*TEM, *Amp*C and *Erm*B), we used Color Perpetual Taq PCR Master Mix (2×) (E2745, EURx Ltd., Gdansk, Poland) and the following sequences (Table 1). PCR program included the following: initial denaturation (5 min at 95 °C); 30 cycles of denaturation (1 min at 95 °C), annealing (1 min at a different temperature depending on the melting temperature (Tm) and extension (1 min/kb at 72 °C); final extension (7 min at 72 °C) and cooling (∞ at 4 °C).

### 2.8. Metagenomic Sequencing

#### 2.8.1. Amplicon Sequencing of the Bacterial 16S rRNA Gene and the Fungal ITS2 Region

Total genomic DNA was extracted from 5 mL of bioreactor samples using the DNeasy^®^ 96 PowerSoil^®^ Pro Kit (Qiagen, Venlo, The Netherlands), following the manufacturer’s instructions.

#### 2.8.2. Library Preparation for 16S Metagenomic Sequencing

Library preparation for the bacterial 16S rRNA gene sequencing was performed by using the 16S Metagenomic Sequencing Library Preparation Kit (Part # 15,044,223 Rev., Illumina). PCR amplification of the bacterial V3–V4 hypervariable region of the 16S rRNA gene was performed using the widely adopted universal primers: 341F: 5′-CCTACGGGNGGCWGCAG-3′ and 805R: 5′-GACTACHVGGGTATCTAATCC-3′, as described by Klindworth et al. (2013) [63]. For fungi, the ITS2 region was amplified using the universal primers: ITS3: 5′-GCATCGATGAAGAACGCAGC-3′ and ITS4: 5′-TCCTCCGCTTATTGATATGC-3′, following the protocols established by Yurkov et al. (2023) [64]. After PCR amplification, amplicons were purified using magnetic bead-based cleanup (AMPure XP, Beckman Coulter, Brea, CA, USA) and sequenced on the Illumina MiSeq platform (2 × 300 bp paired-end) at Macrogen Inc. (Seoul, Republic of Korea).

#### 2.8.3. Data Preprocessing and ASV Generation

Initial quality assessment of the raw sequencing data was conducted using FastQC and MultiQC to ensure that the sequence data met quality standards. Adapter and primer sequences were removed with Cutadapt (v3.2) [65], and reads were trimmed to 250 bp (forward) and 200 bp (reverse) to ensure uniformity across all samples. High-resolution amplicon sequence variance (ASV) generation was performed using DADA2 (v1.18.0) [66]. Reads with expected error rates ≥2 were discarded. An error model was inferred for each sequencing run, and noisy reads were denoised accordingly. Paired-end reads were merged, and chimeric sequences were removed using the removeBimeraDenovo function in consensus mode. ASVs shorter than 350 bp were filtered out using R (v4.0.3). For downstream microbial community comparisons, samples were normalized via subsampling to the lowest read count using QIIME (v1.9.0) [67].

#### 2.8.4. Taxonomic Assignment

Taxonomic assignment of each ASV was carried out by using BLAST+ (v2.9.0) [68] against the NCBI 16S and NCBI ITS reference databases, with thresholds of query coverage >85% and identity >85%. Only the top hit was retained for each ASV.

#### 2.8.5. Community Composition Visualization

Relative abundances of bacterial and fungal taxa were calculated by normalizing ASV counts to the total sequences in each sample. Taxonomic ranks (genera) were aggregated, and bar plots showing the percentage composition of microbial communities were generated using the phyloseq (v1.34.0) and ggplot2 (v3.3.3) packages in R (v4.0.3).

### 2.9. Statistical Analysis

The results of determining the percentage of degraded cellulose and antibiotic sensitivity were performed in triplicate and presented as average values ± standard deviation (SD), where it is possible. Experimental data were analyzed statistically using ANOVA. Statistical significance was defined as a *p*-value < 0.05.

## 3. Results

### 3.1. Cellulose Biodegradation

**Figure 1:**
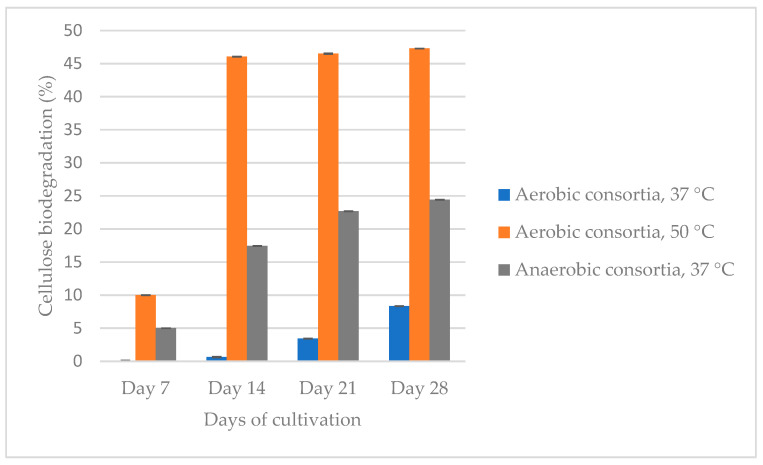
Comparison of the ability of the three isolated and enriched consortia (aerobic at 37 °C (reculture 3) and 50 °C (reculture 4) and anaerobic at 37 °C (reculture 2) to degrade cellulose for 28 days.

We compared three isolated and cultured at different conditions microbial consortia—aerobic at 37 °C and 50 °C and anaerobic at 37 °C (Figure 1).

**Figure 2:**
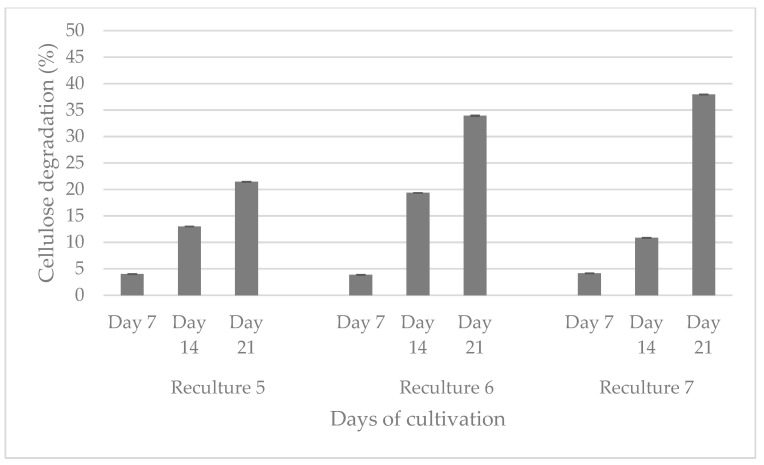
Comparison of the ability of three anaerobic recultures to degrade cellulose for 21 days.

The aerobic consortium cultivated at 50 °C degrades cellulose to the greatest extent (47.3%) for 28 days, followed by the anaerobic (24.4%) and aerobic at 37 °C (8.4%) populations. Between the 21st and 28th day, the difference in the percentage of cellulose degradation is not more than 5%. Based on this result, we decided that the recultivation should be carried out on the 21st day so that the consortia be able to preserve their cellulolytic activity and to continue biodegradation of the filter paper. The aerobic population, which was cultured at 37 °C, degraded cellulose up to the reculture 5, while that at 50 °C degraded cellulose up to the reculture 4. The anaerobic consortium degraded filter paper by the reculture 10. It is important to note that it is not economically viable to maintain a high temperature like 50 °C, and therefore, we decided to cease the experiments with this community. Figure 2 shows the anaerobic biodegradation of cellulose from three microbial recultures (recultures 5–7).

An increase in the percentage of degraded cellulose is observed upon inoculation on day 21, from 21.45 to 37.94%.

### 3.2. Antimicrobial Resistance

We performed a screening for antimicrobial resistance of aerobic and anaerobic consortia, which were cultivated on PCS agar and BHA at 37 °C, against 41 antibiotics listed in Table 2.

The results demonstrated that the anaerobic population was more resistant (41.5%) to clinically applied antibiotics (100%), compared to aerobes (29.3%). Aerobic and anaerobic consortia were resistant to oxacillin, ceftazidime, ceftolozane/tazobactam, cefepime, doripenem, aztreonam, nalidixic acid, streptomycin, bacitracin, colistin (methanesulfonate) and lincomycin. Aerobics were also resistant to ceftazidime-avibactam, while the anaerobic population was also resistant to pefloxacin, levofloxacin, kanamycin, rifampin, novobiocin and trimethoprim/sulfamethoxazole.

### 3.3. Detection of Cellulose-Specific Genes and Genes for Antimicrobial Resistance

**Figure 3:**
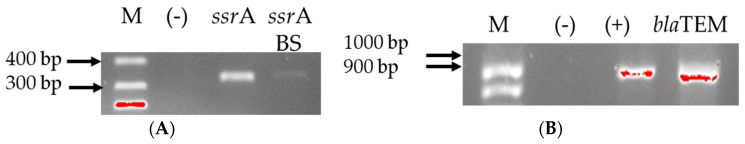
Gel electrophoresis for genes for cellulolytic activity (**A**) and for antibiotic resistance (**B**). Black lines designate non-adjacent samples. *Legend:* M—marker; (+)—positive control; (−)—negative control.

We performed conventional PCR for the detection of genes related to cellulose biodegradation with primers, specific for *Clostridium cellulovorans* and *Bacillus subtilis* (Figure 3A). Additionally, multiplex PCR was carried out to prove the presence of chromosomal and plasmid-encoded genes for antimicrobial resistance in *Escherichia coli* (Figure 3B). The experiment was carried out in order to check whether previous conjugation with *E. coli* has given the consortium bacteria antimicrobial resistance.

Moreover, we tested the anaerobic population after lyophilization, which again degraded the filter paper into pieces, i.e., the activity was retained, for carrying a total of eight genes for cellulolytic activity. They were positive for transfer-messenger RNA gene in *C. cellulovorans* and *B. subtilis*. The two consortia were checked for the presence of nine genes for antibiotic resistance. Only the *bla*TEM β-lactam-resistant gene was detected among the anaerobic cultivated consortium.

### 3.4. Metagenome Analysis

**Figure 4:**
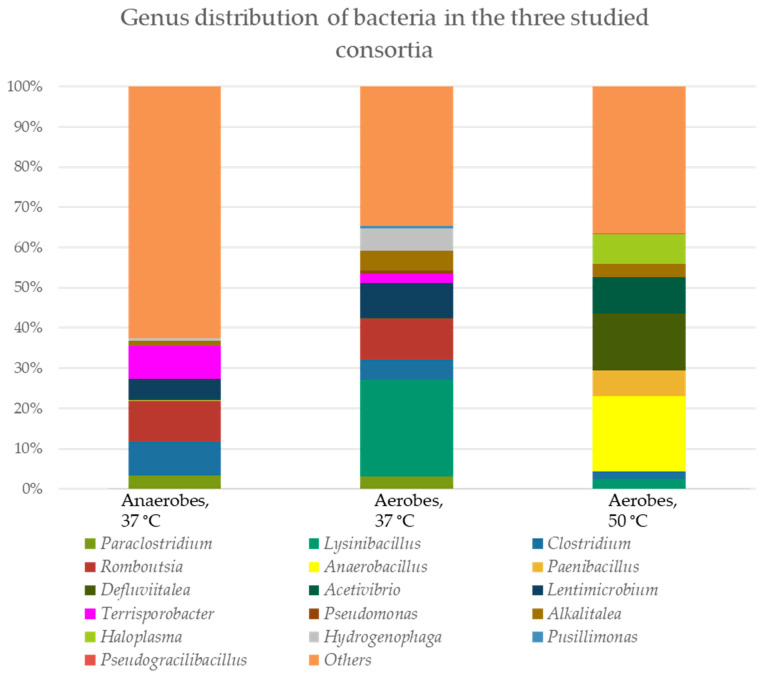

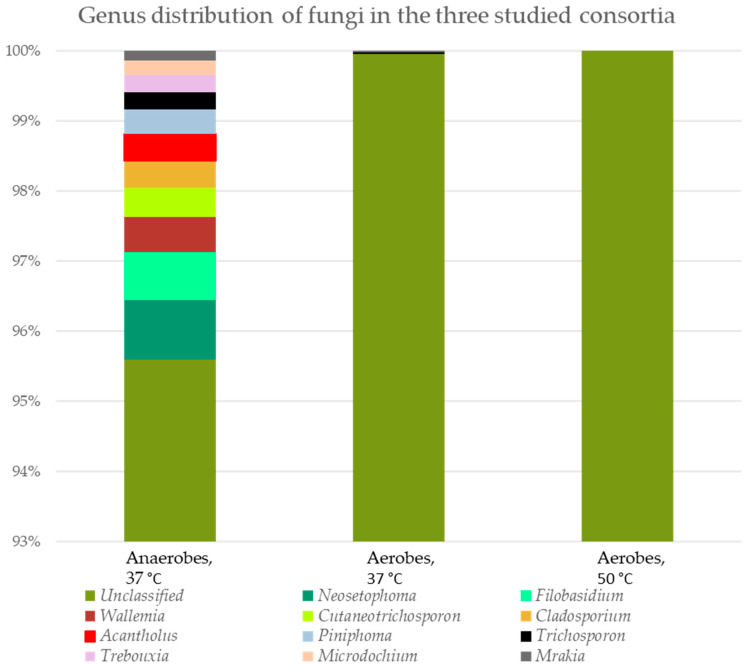
Genus distribution of microorganisms in the three tested consortia. The percent domination for bacteria is presented as cDNA, and for fungi, as amplicon sequence variance (ASV).

The bacterial and fungal genus distributions in the three consortia (aerobes at 37 °C and 50 °C, and anaerobes at 37 °C) were studied (Figure 4).

A similarity of the representative species between the anaerobic and aerobic populations cultivated at 37 °C from the genera *Paraclostridium* (3.26% and 3.05% cDNA, respectively), *Clostridium* (8.53% and 4.82% cDNA, respectively), *Romboutsia* (10.11% and 10.20% cDNA, respectively), *Lentimicrobium* (4.91% and 8.58% cDNA, respectively), *Terrisporobacter* (8.14% and 2.25% cDNA, respectively) and *Hydrogenophaga* (0.65% and 5.63% cDNA, respectively) was found. Only genus *Alcalitalea* is represented in the three populations (between 3.34 and 4.88% cDNA in the two aerobic consortia and 1.09% cDNA in the anaerobic consortium). In the aerobic population cultured at 50 °C dominated *Haloplasma* spp. (7.49% cDNA), *Acetivibrio* spp. (8.86% cDNA), *Defluvitalea* spp. (14.26% cDNA) and *Paenibacillus* spp. (6.30% cDNA). Between aerobes at 37 °C and 50 °C, there were also similarities (*Lysinibacillus* spp. − 24.19% and 2.57%, respectively).

Interestingly, fungal representatives were also found in the three consortia, most of which were unclassified. Only two genera were found in the aerobic and anaerobic consortia at 37 °C—*Trichosporon* (0.02% and 0.24% ASV, respectively) and *Mrakia* (0.02% and 0.14% ASV, respectively). The diversity of different fungal representatives in the anaerobic consortium is impressive. We found also species in the anaerobic population from the genera *Microdochium* (0.21% ASV), *Trebouxia* (0.25% ASV), *Piniphoma* (0.35% ASV), *Acantholus* (0.38% ASV), *Cladosporium* (0.38% ASV), *Cutaneotrichosporon* (0.42% ASV), *Wallemia* (0.50% ASV), *Filobasidium* (0.68% ASV) and *Neosetophoma* (0.85% ASV).

## 4. Discussion

### 4.1. Cellulose Biodegradation

The present study represents a combined approach of isolation, enrichment and analysis, which aims to obtain an active microbial community that demonstrates and retains its cellulose-degrading capacity for a long period of time, as well as to be free from resistant pathogens. The enrichment process contributes to the selectivity of microorganisms to the cultivation conditions, which has an impact on the composition and structure of each consortium [69]. Of course, the main influence is the raw material from which the consortium is isolated, as well as the source of cellulose. In this regard, researchers obtain different percentages and rates of cellulose biodegradation. For example, Feng et al. (2011) isolated a microbial consortium from the soil, and after enrichment, it can degrade more than 51% of non-sterilized raw corn stover powder or 81% of non-sterilized filter paper within 8 days at 40 °C under facultative anoxic conditions [69]. Another consortium reduced the corn straw weight by 48% compared to that at 24 days [18]. Gad et al. (2024) [19] isolated single colonies from different cellulose sources, examined their cellulolytic potential, and combined the most active colonies into two consortia that degraded filter paper by 46.15% and 43.76% in 2 days. The first consists of *Cellulomonas uda* and *Pseudomonas citronellolis*, and the second of *C. uda* and *P. jinjuensis*. Li et al. (2024) [20] also isolated a single fungal species and checked it for cellulolytic activity. The fungal strain *Fusarium fujikuroi* showed a significantly higher degradation rate of 37.64% after 30 days of fermentation compared to the other single strains. The most cellulose-degrading five strain combinations were (1) *Neurospora intermedia* and *Lysinibacillus macrolides* (40.05%), (2) *Streptomyces* sp. and *Bacillus subtilis* (40.33%), (3) *Gibberella moniliformis* and *L. macrolides* (39.85%), (4) *G. moniliformis* and *B. subtilis* (38.78%) and (5) *Fusarium fujikuroi* and *B. subtilis* (37.10%). Gupta et al. (2012) demonstrated the capacity of eight cellulose-degrading bacteria isolated from the guts of a termite, snail, caterpillar and bookworm to degrade filter paper (55–65.7%) in 3 days by a gravimetric method [21]. The microbial consortium isolated from the hindgut of *Holotrichia parallela* larvae degraded about 83.1% of rice straw within three days [22]. Our results showed that reculture 4 of the aerobic consortium, which was isolated from a working methanogenic bioreactor and cultivated at 50 °C, degraded filter paper by 46.05% for 14 days, after which, at subculture 5, its activity stopped (Figure 1). It must be taken into account that maintaining such a high temperature requires additional energy, which is not economically beneficial. In comparison, anaerobes (reculture 2) that were grown at 37 °C degraded cellulose 17.44% for 14 days and 24.4% for 28 days. After enrichment by subculturing, the cellulose degradation reached 37.94% for 21 days (Figure 2, see the visualization of the results in the Supplementary Materials).

### 4.2. Long-Term Storage

An important issue that should be taken into account is the duration of time for which single bacterial species or bacterial consortia, retain their cellulolytic activity. In our case, it is important to note that anaerobes degraded cellulose substrate up to the 10th recultivation during approximately one year period. On the other hand, aerobes degraded the cellulose relatively weakly (8.35% for 28 days, Figure 1).

### 4.3. Antimicrobial Resistance and Genes for Antibiotic Resistance

Non-plant pathogenic endophytic bacteria can enhance nitrogen nutrition, stimulate plant growth and occasionally pose a threat to human and animal health [70]. When searching for cellulose-degrading consortia or single colonies for real-world applications, their antimicrobial resistance must also be assessed. Therefore, we determined the antibiotic susceptibility analysis of the aerobic and anaerobic communities cultured at 37 °C (Table 2). The anaerobic population was more resistant (41.5%) to clinically applied antibiotics, compared to aerobes (29.3%). Aerobic and anaerobic consortia were resistant to penicillins (oxacillin), cephalosporins, carbepenemes (doripenem), monobactames, fluoroquinolones, aminoglycosides (streptomycin) and other agents such as bacitracin, colistin (methanesulfonate) and lincomycin. Aerobes also exhibited resistance to ceftazidime-avibactam, whereas the anaerobic population exhibited resistance to pefloxacin, levofloxacin, kanamycin, rifampin, novobiocin and trime-thoprim/sulfamethoxazole. Interestingly, the two tested consortia showed resistance to oxacillin and were sensitive to all other antibiotics in the penicillin group. The resistance to oxacillin is common, especially among enteric Gram-negative bacilli [71]. For example, *Enterobacterales* such as *Citrobacter freundii* [72,73], *Yersinia enterocolitica* [74,75], *Morganella morganii* [76], *Proteus vulgaris* [77,78], etc. and non-fermenters, such as *Pseudomonas aeruginosa* [79], *Acinetobacter* spp. [80], etc., which can enter a biogas digester, are intrinsically resistant to a large spectrum of antibiotics, including oxacillin [81]. The same applies to pathogens such as *Neisseria* spp. [82], *Campylobacter* spp. [83] and *Helicobacter pylori* [84]. *Bacillus subtilis* can also be resistant to oxacillin but the mechanism has not yet been elucidated [85], compared with the another Gram-positive bacteria, which encode altered penicillin-binding protein 2a (*Staphylococcus aureus* [86], *Enterococcus faecalis* [87], etc.). Widespread bacterial β-lactamases, such as the TEM and SHV enzymes, encoded by the *bla*TEM and *bla*SHV genes, according to literature, have a weak ability to degrade oxacillin but the drug is not their typical substrate [88]. The β-lactamases that degrade oxacillin are the class D β-lactamases, or oxacillinases, for example, the OXA and PSE enzymes. The OXA enzymes are most commonly found in *P*. *aeruginosa*, *A*. *baumannii* and *Klebsiella pneumoniae*. Most of these OXA enzymes are not termed extended-spectrum β-lactamases (ESBLs) because they lack hydrolyzing capabilities. Group 4 β-lactamases are incompletely characterized enzymes. They have been found to hydrolyze cloxacillin, which shares its core structure with oxacillin. They have been found in *Clostridium butyricum* (with weak cloxacillin-hydrolyzing activity) and *Pseudomonas* spp., *Escherichia coli* and other bacterial species [89,90,91,92]. Interestingly, from all tested genes for antibiotic resistance we found *bla*TEM in the anaerobic population (Figure 3B). Some authors report that they found oxacillin-resistant *S. aureus* and are *mec*A or *mec*C free. Their β-lactamase enzyme, BlaZ, was found to hydrolyze oxacillin significantly more than BlaZ from methicillin-sensitive *S. aureus*, thus acting as an ESBL [93,94]. *Clostridium* spp. are not extensively studied for oxacillin resistance or when studied, they are sensitive [95,96].

### 4.4. Genes for Cellulolytic Activity and Stress Proteins

Bacteria are constantly exposed to stressful conditions—exposure to antibiotics, nutrient depletion and oxidative stress. In order to adapt to these conditions, the organism must be able to quickly change its gene expression program in order to combat the stress. RNA-binding proteins are essential for controlling gene expression during adaptive responses because they mediate co- and post-transcriptional regulation [97]. In the anaerobic consortium, we found two genes for RNA-specific binding protein (Figure 3A)—*ssr*A (for *Clostridium* sp.) and *ssr*A BS (for *Bacillus subtilis*), and no gene for cellulolytic activity (*cbp*A, *exg*S, *eng*H, *eng*E, *eng*M and *xyn*A). The *ssr*A gene encodes a transfer-messenger RNA (tmRNA) that functions both as a tRNA and mRNA to rescue ribosomes, and is found in all sequenced prokaryotic genomes, indicating its biological importance in the trans-translation system [98]. *Ssr*A is essential for the growth of *B. subtilis* at 52 °C or under cadmium or ethanol stress conditions [49]. The presence of this gene in our sample can be explained by nutrient depletion, since the sample was taken on the 14th day of the new culture after lyophilization, when we observe an increase in the rate of cellulose degradation or due to the lyophilization process itself.

### 4.5. Cellulose-Degrading Bacterial Species

The aerobic consortium cultured at 50 °C was characterized by the highest percentage of cellulose degradation (47.3%, Figure 1), followed by the anaerobes (39.94%, Figure 2), which degraded up to the 10th reculture inclusive. In the aerobes, representatives of the genera *Defluvitalea* (14.26% cDNA) and *Anaerobacillus* (18.68% cDNA) dominated (Figure 4). The Bacillota (synonym of Firmicutes) include strict or facultative anaerobic bacteria of the genera *Clostridium* and *Defluvitalea*, as well as *Ruminiclostridium*, whose members degrade complex carbohydrates such as cellulose, xylan and cellobiose [99]. The presence of *Defluvitalea* spp. in our probe is not unusual. They have been reported to be involved in the fermentation of saccharides under thermophilic conditions in the biogas production bioreactor, with no occurrence above 55 °C [100]. *Defluviitalea* spp. were found to be up to 34% present in cellulose-enriched cultures or to be equivalent to 90% of the relative abundance in cellulose-mixed isolates derived from thermophilic laboratory biogas fermenters [101]. The genus *Anaerobacillus* contains anaerobic or aerotolerant Gram-positive rods that grow through fermentative or anaerobic respiration in obligate or moderately alkaliphilic and halophilic environments [102]. A product of alkaline hydrolysis of cellulose found in pulp wastewater streams is isosaccharinate [103], which is characterized by being degraded by *Anaerobacillus* spp. [102]. There are a lot of data in the literature on the ability of Acetivibrio to degrade cellulose. *Acetivibrio cellulolyticus* and *Clostridium saccharolyticum* were isolated before from the methanogenic cellulose-enriched culture, with the two representatives competing with each other. *A. cellulolyticus* provided assimilable sugars (including glucose) which *C*. *saccharolyticum* converted to acetic acid and H_2_, known substrates for methanogens, and ethanol and lactic acid, known substrates for sulfate-reducing bacteria [104]. The presence of *Lysinibacillus* spp. in both aerobic populations, with the number of cDNAs being higher at 37 °C (24.19% cDNA) compared to 50 °C (2.57% cDNA), is striking (Figure 4). This genus degraded xylan [105]. *Lysinobacillus macrolides*, *L. sphaericus*, *Bacillus subtilis and Paenibacillus lautus* participate in corn straw composting by a microbial consortium [20]. A microbial consortium composed of *Parabacteroides*, *Alcaligenes*, *Lysinibacillus*, *Sphingobacterium* and *Clostridium* showed efficient degradation of rice straw, in which cellulose, hemicelluloses and lignin lost 71.7%, 65.6% and 12.5%, respectively, of their weight, after cultivation for 20 days at 15 °C. By proteomics, the lignocellulose-degrading enzyme system was analyzed in *Clostridium* spp. (β-glucosidase-related glycosidases, α-L-arabinofuranosidase, xylanase and type IIS restriction enzyme protein) and in *Lysinibacillus saudimassiliensis* (endoglucanase precursor and catalase) [106]. The genus *Romboutsia* was represented by almost equal numbers of cDNAs in the anaerobic (10.11%) and aerobic (10.20%) consortia cultured at 37 °C, suggesting them as facultative anaerobic bacteria (Figure 4). A recent study shows that this genus participates in the lignocellulosic anaerobic co-digestion process and demonstrates its ability to degrade carbohydrates [107], including cellulose, and ferment them into volatile fatty acids, H_2_ and CO_2_ [108]. According to Lu et al. (2025) [109], the cellulolytic bacterial composition changed during the progress of the degradation process. At the beginning of the process, *Paraclostridium* spp. are predominant and possibly initiate the lignocellulose degradation, after which *Clostridium* spp. and *Terrisporobacter* spp. are involved after growing rapidly. *Lysinibacillus* spp. grow after the third day from the start of the process. Fang et al. (2021) reported that *Paraclostridium* spp., which are found in our aerobic and anaerobic consortia cultured at 37 °C (Figure 4), showed a suppressive ability to fluoroquinolone antibiotic [110], which explains the resistance shown in our studies (Table 2) and to some extent why we have not been able to prove an antibiotic resistance gene to this group of antibiotics (Figure 3B). *Lentimicrobium* spp., which participated twice as much in the aerobic population (8.58% cDNA) as in the anaerobic one (4.91%) (Figure 4), were previously isolated from methanogenic granular sludge. The authors mentioned that *L. saccharophilum* can ferment glucose to acetate, malate, propionate, formate and H_2_ [111].

## 5. Conclusions

In summary, our study represents a comparative analysis of the ability of three microbial populations isolated from a working methanogenic bioreactor to maintain their cellulose-degrading capacity through cultivation and adaptation to different environmental conditions—aerobic at 37 °C and 50 °C and anaerobic at 37 °C. High biodegradation of cellulose was achieved in 21 days under aerobic conditions at 50 °C (46.5%) and anaerobic conditions at 37 °C (37.9%). The latter degraded filter paper into pieces as a cellulose source up to 10 subcultures. The antimicrobial resistance to 42 clinically applied antibiotics was determined. The anaerobic population was found to be more resistant (41.5%), compared to aerobes (29.3%) cultured at 37 °C, which degraded cellulose to a weaker degree, 8.35% in 28 days. We proved the presence in the anaerobic population of *ssr*A and *ssr*A BS genes that are transfer-messenger RNA genes and play a key role in environmental stress factors, as well as *bla*TEM, a gene for β-lactamase, encoded on the plasmid. By metagenomics analysis, we investigated the representative genus in the three tested consortia. In the anaerobic population, the dominant species are from the genera *Roumboutsia*, *Clostridium*, *Terrisporobacter*, *Lentimicrobium* and *Paraclostridium*; in the aerobic consortium cultured at 37 °C, they are *Lysinibacillus*, *Roumboutsia*, *Lentimicrobium*, Hydrogenophaga, *Alkalitalea*, *Clostridium*, *Paraclostridium* and *Terrisporobacter*; and in the same conditions, but at 50 °C, they are *Anaerobacillus*, *Defluvitalea*, *Acetivibrio*, Haloplasma, *Paenibacillus*, *Alkalitalea*, *Lysinibacillus* and *Clostridium.* The reported cellulolytic activity and the long period of its preservation are important points from a technological point of view. We believe that our research will contribute to environmental protection and cost-effective management of lignocellulose waste.

## Figures and Tables

**Table 1:** List of the primers used in this study.

Gene	Enzyme Encoded	Sequences	Tm	Reference
*Cbp*A F	Cellulose-binding protein	ATGCAAAAAAAGAAATCGCTG	62.9 °C	[42,43,44]
*Cbp*A R		GGTTGATGTTGGGCTTGCTGTTTC	52.3 °C	
*Eng*H F	Endoglucanase H	GTGTTT AACATATCTAAGAAAAAA	53.1 °C	[45]
*Eng*H R		CTACTGTGATAAAAGTAGTTTC	49.3 °C	
*Eng*M F	Endoglucanase M	ATGAATAGAAAAAAAATAACAGC	55.1 °C	[46]
*Eng*M R		TTATGCAAGCAGTTGTTTCTTTA	60.4 °C	
*Eng*E F	Endoglucanase E	ATGAAGAAGAGAAACAGAATA	52.2 °C	[47]
*Eng*E R		TTATATTGCTTTTTTTAAGAATGC	57.4 °C	
*Exg*S F	Exoglucanase S	ATGAGAAAAAGATTAAATAAG	49.0 °C	[45]
*Exg*S R		TTAAGCAAGAAGTGCTTTCT	56.1 °C	
*Ssr*A BS F	Transfer-messenger RNA gene in *B. subtilis*	GGGGACGTTACGGATTCGACA	70.0 °C	[48,49]
*Ssr*A BS R		GTATGGAGACGGTGGGAGTCGAA	70.2 °C	
*Ssr*A F	Transfer-messenger RNA gene in *C. cellulovorans*	GGGGGTGTACTTGGTTTCGA	65.9 °C	[50]
*Ssr*A R		TGGTGGAGGTGAGGGGTG	67.5 °C	
*Xyn*A F	Xylanase	ATGAAACAAAAAATGAGGATAGTT	58.6 °C	[51]
*Xyn*A R		TTAGAATGCACCATTTAACAT	56.2 °C	
ESBLs-TEM F	Extended-spectrum plasmid-mediated β-lactamases	GGGGATGAGTATTCAACATTTCC		[52]
ESBLs-TEM R		GGGCAGTTACCAATGCTTAATCA		
*Qnr*A F	Plasmid-mediated quinolone resistance protein	GGGTATGGATATTATTGATAAAG	55.0 °C	[53]
*Qnr*A R		CTAATCCGGCAGCACTATTTA	60.7 °C	
*Qnr*B F		GATCGTGAAAGCCAGAAAGG	63.6 °C	[54]
*Qnr*B R		ACGATGCCTGGTAGTTGTCC	63.9 °C	
*Qnr*B60 F		ATGGCTCTGGCATTAATTGGCG	62.1 °C	[55]
*Qnr*B60 R		TTAGCCAATGACAGCGATGCC	61.2 °C	
*Aac*(3)-IV F	Plasmid-encoded aminoglycoside acetyltransferase	CTTCAGGATGGCAAGTTGGT	64.0 °C	[56]
*Aac*(3)-IV R		TCATCTCGTTCTCCGCTCAT	64.9 °C	
*Bla*SHV F	β-lactamase encoded on the plasmid	TCGCCTGTGTATTATCTCCC	61.5 °C	[57]
*Bla*SHV R		CGCAGATAAATCACCACAATG	62.9 °C	
*Bla*TEM F		TCGGGGAAATGTGCGCG	71.9 °C	[58]
*Bla*TEM R		TGCTTAATCAGTGAGGCACC	62.8 °C	
*BlaCMY F*	class C β-lactamase encoded on the plasmid	TTTCTCCTGAACGTGGCTGGC	70.1 °C	[59]
*BlaCMY R*		TGGCCAGAACTGACAGGCAAA	70.7 °C	
*Amp*C F	Cephalosporinases encoded on the chromosomes	AATGGGTTTTCTACGGTCTG	60.4 °C	[60]
*Amp*C R		GGGCAGCAAATGTGGAGCAA	70.5 °C	
*Amp*C F		GTGACCAGATACTGGCCACA	60.5 °C	[61]
*Amp*C R		TTACTGTAGCGCCTCGAGGA	60.5 °C	
*Erm*B F	Plasmid-encoded erythromycin-resistant methylase	GAAAAAGTACTCAACCAAATA	52.7 °C	[62]
*Erm*B R		AATTTAAGTACCGTTAC	43.0 °C	
*Erm*B F		GCATTTAACGACGAAACTGGC	60.1 °C	[61]
*Erm*B R		GACAATACTTGCTCATAAGTAATGGT	61.7 °C	

**Table 2:** Antimicrobial resistance of aerobic and anaerobic consortia, isolated from methanogenic bioreactor and cultured on two different media at 37 °C.

Drug Class	Antibiotic	Aerobic Consortium		Anaerobic Consortium	
		**PCS**	**BHA**	**PCS**	**BHA**
Penicillins	Amoxicillin	S	S	S	S
	Amoxicillin/-clavulanic acid	S	S	S	S
	Ampicillin	S	S	S	S
	Carbenicillin	S	S	S	S
	Oxacillin	**R**	**R**	**R**	**R**
	Penicillin G	S	S	S	S
	Piperacillin	S	S	S	S
	Piperacillin/-tazobactam	S	S	S	S
	Ticarcillin	S	S	S	S
	Ticarcillin/clavulanic acid	S	S	S	S
Tetracyclines	Doxycycline	S	S	S	S
	Doxycycline HCl	S	S	S	S
	Tetracycline	S	**I**	S	S
Cephalosporins	Cefamandole	S	S	S	S
	Ceftazidime	S	**R**	**R**	S
	Ceftazidime-avibactam	**R**	**R**	S	S
	Ceftolozane/tazobactam	S	**R**	**R**	**R**
	Cefepime	**R**	**R**	**I**	**R**
Carbapenems	Doripenem	**R**	**R**	**R**	**R**
	Imipenem	S	S	S	S
	Meropenem	S	S	S	S
Monobactams	Aztreonam	S	**R**	**R**	**R**
Fluoroquinolones	Ciprofloxacin	S	S	**I**	S
	Pefloxacin	S	S	**R**	**R**
	Levofloxacin	S	S	**R**	**R**
	Nalidixic acid	**R**	**R**	**R**	S
Aminoglycosides	Amikacin	S	S	S	**I**
	Gentamycin	S	S	S	S
	Kanamycin	**I**	S	S	**R**
	Streptomycin	**R**	**R**	**R**	S
	Tobramycin	**I**	S	S	S
Glycopeptides and lipoglycopeptides	Vancomycin	S	S	S	S
Macrolides	Erythromycin	S	**I**	S	S
	Clarithromycin	S	S	S	S
	Rifampin	S	S	S	**R**
Other agents	Chloramphenicol	S	S	S	S
	Bacitracin	**R**	**R**	**R**	**R**
	Colistin (methanesulfonate)	**R**	**R**	**R**	**R**
	Lincomycin	**R**	**R**	**R**	**R**
	Novobiocin	S	S	**R**	**R**
	Trimethoprim/sulfamethoxazole	S	S	**R**	**R**

Legend: S-susceptible, I-intermediately resistant and R-resistant.

## Data Availability

The original contributions presented in this study are included in the article. Further inquiries can be directed to the corresponding author.

## Supplementary Materials

The following supporting information can be downloaded at: https://www.mdpi.com/article/10.3390/genes16050551/s1.
