# Supplementary figures and images for: Isolation, Enrichment and Analysis of Aerobic, Anaerobic, Pathogen-Free and Non-Resistant Cellulose-Degrading Microbial Populations from Methanogenic Bioreactor
Source: Genes (Basel). 2025 Apr 30;16(5):551. doi: 10.3390/genes16050551 (PMC12111770; doi:10.3390/genes16050551)

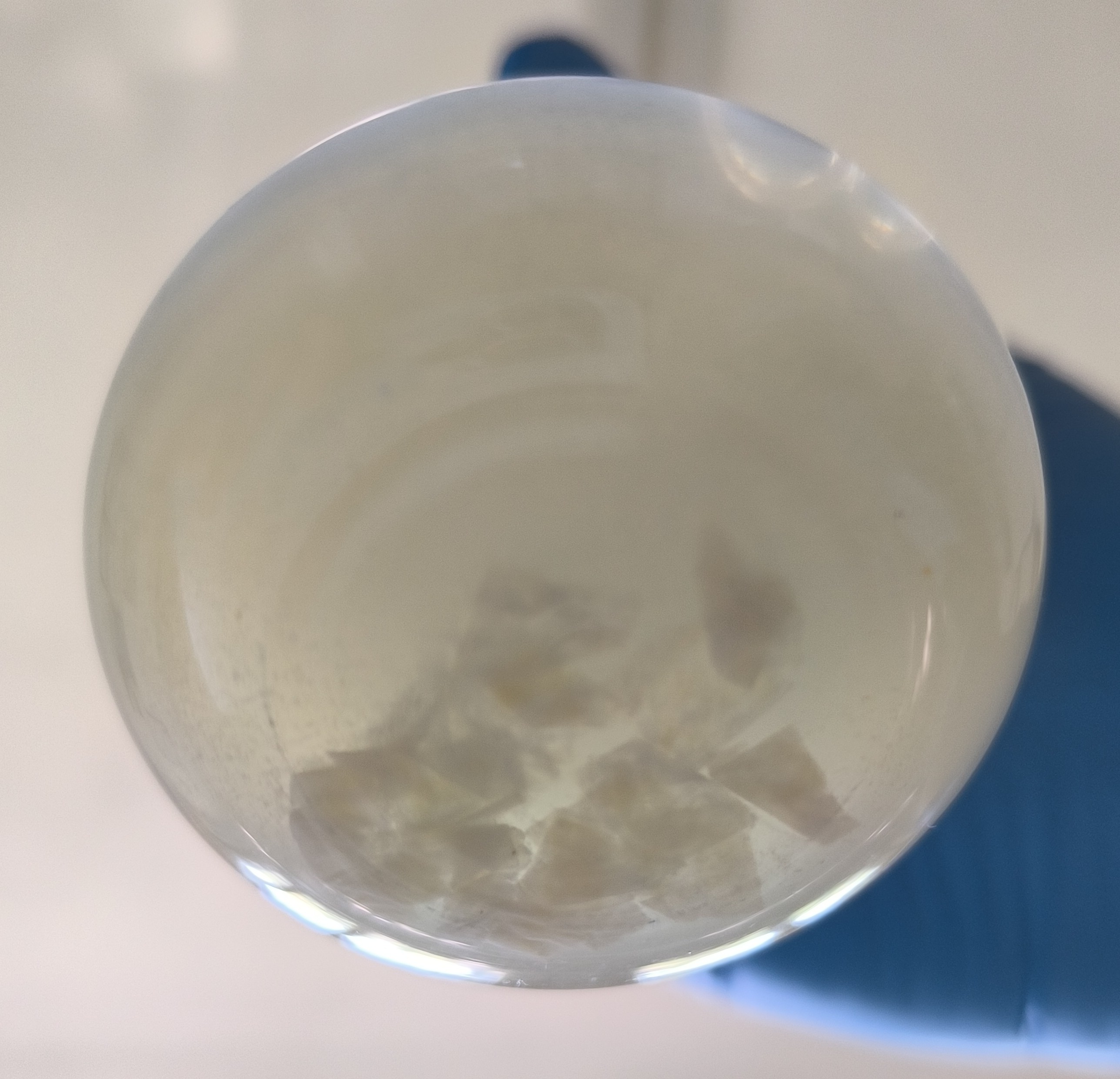

Supplement: Supplementary file 1 [file genes-16-00551-s001.zip › PCS medium supplemented with filter paper pieces and anaerobic consortium for 14 days_flask bottom view.jpg]

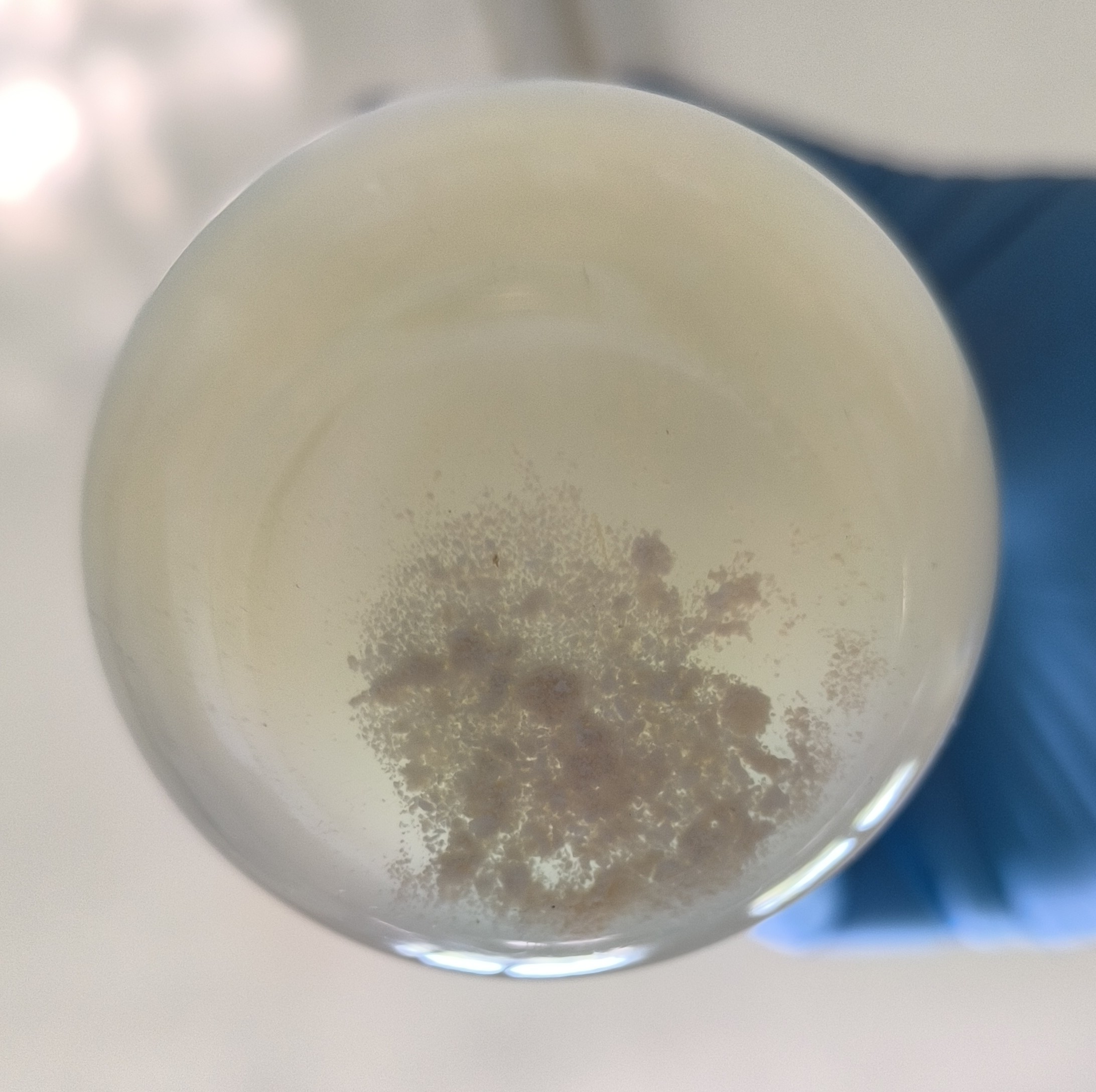

Supplement: Supplementary file 1 [file genes-16-00551-s001.zip › PCS medium supplemented with filter paper pieces and anaerobic consortium for 21 days_flask bottom view.jpg]

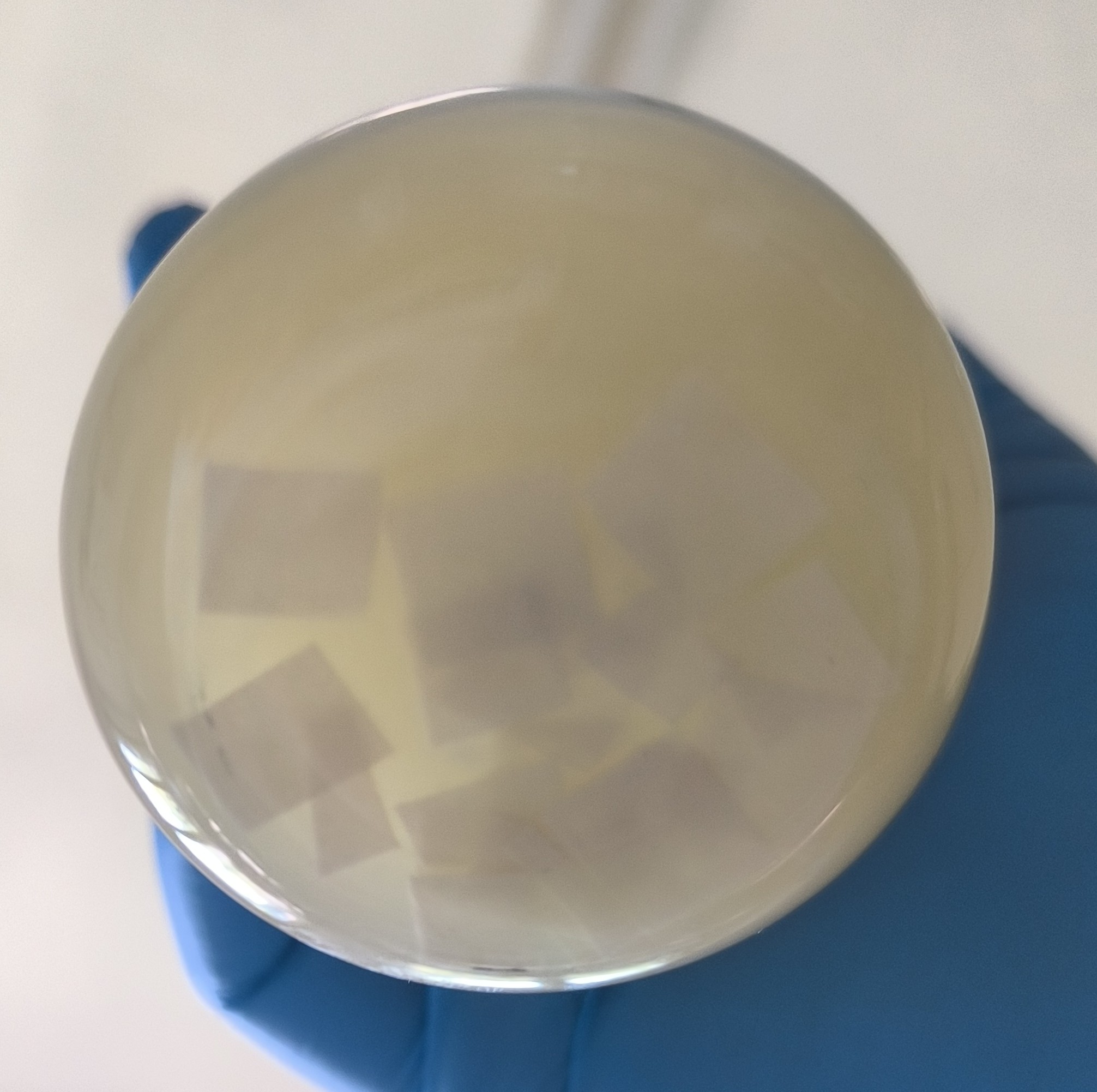

Supplement: Supplementary file 1 [file genes-16-00551-s001.zip › PCS medium supplemented with filter paper pieces and sterilized anaerobic consortium for 28 days_flask bottom view.jpg]
